# Negative symptoms and social cognition as mediators of the relationship between neurocognition and functional outcome in schizophrenia

**DOI:** 10.3389/fpsyt.2024.1333711

**Published:** 2024-01-31

**Authors:** Giulia M. Giordano, Pasquale Pezzella, Armida Mucci, Stephen F. Austin, Andreas Erfurth, Birte Glenthøj, Alex Hofer, Jan Hubenak, Jan Libiger, Ingrid Melle, Mette Ø. Nielsen, Janusz K. Rybakowski, Pawel Wojciak, Silvana Galderisi, Gabriele Sachs

**Affiliations:** ^1^ Department of Psychiatry, University of Campania “Luigi Vanvitelli”, Naples, Italy; ^2^ Psychiatric Research Unit, Region Zealand Psychiatry, Slagelse, Denmark; ^3^ 6th Psychiatric Department, Otto-Wagner-Spital, Vienna, Austria; ^4^ Center for Neuropsychiatric Schizophrenia Research (CNSR) and Center for Clinical Intervention and Neuropsychiatric Schizophrenia Research (CINS), Mental Health Center Glostrup, Glostrup, Denmark; ^5^ Department of Clinical Medicine, Faculty of Health and Medical Sciences, University of Copenhagen, Copenhagen, Denmark; ^6^ Medical University Innsbruck, Department of Psychiatry, Psychotherapy, Psychosomatics and Medical Psychology, Division of Psychiatry I, Innsbruck, Austria; ^7^ Department of Psychiatry, Charles University, Faculty of Medicine and University Hospital Hradec Králové, Hradec Králové, Czechia; ^8^ NORMENT Centre, Institute of Clinical Psychiatry, University of Oslo and Oslo University Hospital, Oslo, Norway; ^9^ Department of Adult Psychiatry, Poznan University of Medical Sciences, Poznan, Poland; ^10^ Department of Psychiatry and Psychotherapy, Medical University of Vienna, Vienna, Austria

**Keywords:** schizophrenia, negative symptoms, expressive deficit, motivational deficit, emotion recognition, processing speed, mediation analysis

## Abstract

**Introduction:**

In this study we assessed the contribution of psychopathology, including the two domains of negative symptoms (motivational deficit and expressive deficit), processing speed as an index of neurocognition, and emotion recognition, as an index of social cognition, to poor functional outcomes in people with schizophrenia.

**Methods:**

The Positive and Negative Syndrome Scale was used to evaluate positive symptoms and disorganization and the Brief Negative Symptom Scale to assess negative symptoms. The Symbol Coding and the Trail Making Test A and B were used to rate processing speed and the Facial Emotion Identification Test to assess emotion recognition. Functional outcome was assessed with the Personal and Social Performance Scale (PSP). Regression analyses were performed to identify predictors of functional outcome. Mediation analyses was used to investigate whether social cognition and negative symptom domains fully or partially mediated the impact of processing speed on functional outcome.

**Results:**

One hundred and fifty subjects from 8 different European centers were recruited. Our data showed that the expressive deficit predicted global functioning and together with motivational deficit fully mediated the effects of neurocognition on it. Motivational deficit was a predictor of personal and social functioning and fully mediated neurocognitive impairment effects on the same outcome. Both motivational deficit and neurocognitive impairment predicted socially useful activities, and the emotion recognition domain of social cognition partially mediated the impact of neurocognitive deficits on this outcome.

**Conclusions:**

Our results indicate that pathways to functional outcomes are specific for different domains of real-life functioning and that negative symptoms and social cognition mediate the impact of neurocognitive deficits on different domains of functioning. Our results suggest that both negative symptoms and social cognition should be targeted by psychosocial interventions to enhance the functional impact of neurocognitive remediation.

## 1 Introduction

Schizophrenia is a severe and heterogenous mental disorder (1–3), that has profound effects on the functional outcomes of those living with the disorder (4–13). Major areas of functioning are impaired in subjects with schizophrenia, such as social, vocational, independent living, self-care, interpersonal relationships, everyday life skills and work abilities (4–6, 14–18). Functional outcome in schizophrenia does not depend only on clinical symptoms, but also on several other variables; some of these are related to patient’s personal resources, some others to the environment in which the patient lives and some to the disease (4–6, 15–17, 19, 20). Among personal resources, coping strategies, recovery style, physical status and resilience, were found to affect real-life functioning (21–24); additionally, environmental factors, including poor economic status, support services, and neighborhoods conditions, have been associated with poor functional outcome (17, 25).

In the present article we will focus on disease-related aspects, in particular on neurocognition, social cognition and negative symptoms, since they represent the major predictors of a poor functional outcome (17, 26, 27). However, to date, the recognition, assessment and management of these factors have often been inadequate, and there are limited treatment options for their management (26–35). Moreover, the complex relationships among these predictors and functioning are still poorly investigated. Understanding pathways to real-life functioning of patients with schizophrenia is essential to design new integrated and personalized treatment plans and to improve patient’s quality of life (29, 30).

Neurocognition has been found to be directly or indirectly (through the impact of other variables) associated to two important domains of functioning, i.e., work skills and everyday life skills (4, 5, 9). It is important to emphasize that neurocognition in schizophrenia is characterized by impairments in different cognitive domains such as attention, memory, verbal learning, visual learning, problem solving and processing speed (27, 36). Among these domains, processing speed has been found to be present in most patients. Indeed, in two meta-analyses its effect size was estimated to be between -1.3 and 1.5 (37, 38). Furthermore, impairment in processing speed was not only associated with poor functional outcome at baseline, but also predicted a poor prognosis longitudinally (27, 37, 39–43). Negative symptoms and social cognition seem to be the most important variables that may serve as mediators of the relationship between neurocognition and functional outcome (4–6, 9, 15, 17, 42, 44–49).

Social cognition is another important factor strongly related to the impairment of functioning in people with schizophrenia (4–6, 9, 27, 50–54). This factor represents a multidimensional and complex construct including four domains (55): emotion recognition, social perception, theory of mind (ToM), and attributional bias (4, 27). Impaired emotion recognition is found in all phases of the disease, even in subjects at risk to develop a psychosis and in those with a first-episode psychosis (56–58) and predicts poor real-life functioning, including both community and social functioning (59–61).

According to some studies, social cognition is even more strongly associated to functional outcome than neurocognition; it is associated to work skills and interpersonal relationships directly (4, 9) and with everyday life skills through functional capacity (5, 62). According to other studies, it acts as a mediator between the latter and functional outcome (4–6, 15, 45–47, 59, 63–67), and between specific neurocognitive domains, such as processing speed (65), sustained attention (65) and verbal memory (64) and functional outcome. Social perception has been reported as a mediator between early visual processing and functional outcome (66), ToM as mediator between neurocognition and social competence that, in turn, showed a direct link with self-reported functioning (63), while emotion recognition was found to be a mediator between neurocognition and social functioning in people with schizophrenia and first episode psychoses (68).

Negative symptoms have a direct and indirect impact on patient’s real-life functioning (4–6, 15, 69), and seem to mediate the effect of neurocognition on functioning (42, 47–49, 70). Negative symptoms are heterogeneous and can be categorized into two different domains, motivational deficit and expressive deficit, showing different relationships with functional impairment and possibly subtended by different pathophysiological mechanisms (26, 55, 71, 72). Motivational deficit domain was found to influence the area of interpersonal relationships, both directly and indirectly (mediated by variables such as internalized stigma, resilience and engagement with services), while expressive deficit domain was found to be indirectly and weakly related to real-life functioning by some studies (4). In a network analysis, motivational deficit domain was associated with interpersonal relationships and work skills, and expressive deficit with everyday life skills (5, 6, 73). Furthermore, only baseline motivational deficit domain predicted interpersonal functioning at four-year follow-up, while expressive deficit domain did not predict functioning (9).

Furthermore, as mentioned above, negative symptoms seem to mediate the effect of neurocognition on real-life functioning, at least in part (48). In more recent papers, these symptoms, together with general psychopathology and insight, were associated to cognition and functioning and acted as mediators in the relationship between both cognition and functioning (49, 74, 75), even in individuals at ultrahigh risk for psychosis (76). A 20-year longitudinal study found that negative symptoms had an indirect impact on work functioning through neurocognition (in particular processing speed and general knowledge) (42). The same study in the cross-sectional analysis at baseline found that negative symptoms mediated the impact of neurocognition on functioning (42).

In summary, the evidence accumulated emphasizes the central role of neurocognition, negative symptoms, and social cognition as primary determinants of functional outcome in individuals with schizophrenia. Notably, as explained above, both negative symptoms and deficits in social cognition seem to mediate the relationships between neurocognition and functioning in patients with schizophrenia and even in the early stages of the disease (42, 47, 48, 64–67).

However, most of the studies did not investigate multiple domains of functioning and the two domains of negative symptoms. In fact, some studies investigated only global functioning or work skills, which might be influenced differently by cognitive impairment and negative symptoms with respect to interpersonal and social functioning, or everyday life skills. Negative symptoms were mostly assessed using first-generation scales and particularly the PANSS which provides an inadequate assessment of negative symptoms and particularly of motivational deficit domain (26).

Within this frame, the present study aimed to evaluate the contribution of psychopathology, including the two domains of negative symptoms, as well as of processing speed (as an index of neurocognition), and emotion recognition (as an index of social cognition), to poor functional outcome in people with schizophrenia. In particular, the specific aims of the paper were to examine: 1) the contribution of psychopathology, processing speed and emotion recognition to poor functional outcome; 2) the possible role of negative symptom domains and emotion recognition as mediators in the relationship between processing speed and specific domains of functional outcome. This study was carried out within the main project “European College of Neuropsychopharmacology (ECNP) Network on Schizophrenia Study on the Assessment of Negative Symptoms” (ENSANeS).

## 2 Methods

In this cross-sectional study, participants were recruited from the outpatient and inpatient units of the psychiatric departments of 8 of the 12 European centers involved in the main project (see **Table 1** for the list of centers and the number of patients recruited in each center). Recruitment took place from 31 October 2016 to 15 July 2017. Inclusion criteria were a diagnosis of schizophrenia according to DSM-IV, confirmed by the MINI Neuropsychiatric Interview-Plus (MINI-Plus), and an age between 18 and 65 years. Exclusion criteria were: (a) change in treatment and/or hospitalization due to exacerbation of symptoms in the previous three months; (b) a history of moderate to severe intellectual disability or neurological disease; (c) a history of alcohol and/or substance abuse in the previous six months; (d) current pregnancy, and (e) inability to provide informed consent. The study was conducted according to the ethical standard established in the 1964 Helsinki Declaration. The local ethics committee of each of the participating centers approved the study. All participants signed a written informed consent to participate in the study.

**Table 1:** Centers involved in the study (N and % of subjects recruited).

Centers	*N*	*%*
**C01.** Austria (Innsbruck, W. W. Fleischhacker, A. Hofer)	25	16.7
**C02.** Austria (Vienna, G. Sachs, A. Erfurth)	20	13.3
**C04.** Czech Republic (J. Libiger)	20	13.3
**C05.** Denmark (B. Y. Glenthøj, M. O. Nielsen)	24	16
**C10.** Italy (S. Galderisi, A. Mucci)	25	16.7
**C11.** Norway (I. Melle)	7	4.7
**C12.** Poland (J. Rybakowski)	20	13.3
**C17.** Denmark (S. Fitzgerald Austin – EURONES)	9	6
Total Sample	150	100.0

### 2.1 Assessment

#### 2.1.1 Psychopathology

The Positive and Negative Syndrome Scale (PANSS) is clinician-rated scale, which assesses positive symptoms, negative symptoms, and general psychopathology (77); each item is rated on a seven-point Likert scale. Higher scores indicate greater severity. In the present study, the positive dimension was calculated according to Wallwork and colleagues (78) by summing the scores on the items “delusions” (P1), “hallucinatory behavior” (P3), “grandiosity” (P5), and “unusual thought” (G9); the disorganization dimension was represented by the PANSS item “conceptual disorganization” (P2), to avoid overlap with cognitive functioning as the PANSS disorganization factor includes impairment in abstract thinking and poor attention.

Negative symptoms were assessed using the Brief Negative Symptom Scale (BNSS) (79). The BNSS is a scale developed according to the current conceptualization of negative symptoms, in accordance with the NIMH-MATRICS Consensus Statement on Negative Symptoms. It assesses all domains of the negative psychopathological dimension: blunted affect, alogia, anhedonia, avolition and asociality, as well as an additional aspect, “distress”, which evaluates the absence of the normal experience of distressing and unpleasant emotions (80). The BNSS consists of 13 items organized into six subscales: anhedonia (three items), distress (one item), asociality (two items), avolition (two items), blunted affect (three items) and alogia (two items). The scale provides a total score (sum of the scores on the 13 items) and six subscale scores (sum of the scores on the items in each subscale). For all items higher scores are associated with greater impairment/presence of symptoms. In the present study, the total negative symptom score was calculated by subtracting the “distress” subscale from the total score (81). The motivational deficit domain of negative symptoms was obtained by summing the scores of the anhedonia, asociality and avolition subscales; the expressive deficit domain was obtained by summing the scores of the subscales alogia and blunted affect.

#### 2.1.2 Processing speed

We chose processing speed as an index of neurocognition. This choice stems from the well-established evidence indicating that it is among the most frequently impaired cognitive domains in individuals with schizophrenia. Processing speed deficits are consistently observed in this population and have been linked to various aspects of functional impairment, making it a relevant and representative measure of neurocognitive functioning (37, 38, 82).

Two tests from the Brief Assessment of Cognition in Schizophrenia (BACS) were used to assess processing speed (83, 84): the Symbol Coding and the Trail Making Test (TMT) A and B. The Symbol Coding is a paper and pencil test that requires the subject to write digits corresponding to meaningless symbols below each symbol, according to a key shown on the top of the page, as quickly as possible, in a maximum time of 90 seconds.

The Trail Making Test (TMT) is made up of two parts, A and B. TMT A is a time trial (300 seconds) in which the subject has to combine randomly arranged numbers on a sheet of paper in ascending order. TMT B is a time trial (300 seconds) in which the participant has to combine numbers and letters randomly arranged on a sheet of paper in ascending and alternating order (i.e.: 1-A-2-B-3-C – etc…), thus combining the numbers (from 1 to 13) and the letters (from A to N) alternately. The TMT A and B scores are based on the number of seconds taken to complete the test; the longer the time taken, the worse the performance. A composite score for “processing speed” was obtained by averaging the Z-score (obtained as the difference between the subject’s score and the sample mean divided by the sample standard deviation) on the three tests (TMT A, TMT B and Symbol Coding, for the latter test the raw scores were inverted before calculating the Z-scores, so that for each test higher Z-scores represented higher impairment).

#### 2.1.3 Emotion recognition

The choice to focus on emotion recognition as an index of social cognition stems from the fact that its impairment appears since the early phases of schizophrenia and has been consistently associated with poor real-life functioning (59–61).

The Facial Emotion Identification Test (FEIT) has been used to assess emotion recognition (85). The FEIT consists of black and white photographs of 55 faces of different people, each showing different emotions. The stimuli were presented in a random order. Participants had to identify the emotion presented in the stimulus (happiness, sadness, anger, fear, surprise, neutral, or disgust). One point was awarded for each correct answer and 0 for incorrect answers. The total FEIT score was calculated as the number of correct responses (0–55).

#### 2.1.4 Functional outcome

The Personal and Social Performance Scale (PSP) was used to assess functional outcome (86). The PSP is a semi-structured interview that investigates 4 areas: self-care, socially useful activities, personal and social relationships, and disturbing and aggressive behavior. Each domain is scored from 0 (no impairment) to 5 (very severe). The scores on the four subdimensions can be combined to produce a total score on a 100-point scale, with higher scores indicating better functioning. In our study we focused on the scores for PSP total, PSP socially useful activities and PSP personal and social relationships.

### 2.2 Statistical analyses

For all measures, the z-score was calculated as the difference between the subject’s score and the sample mean divided by the sample standard deviation.

A preliminary correlation analysis was used to verify associations of clinical and cognitive variables with the functional outcome, using Pearson’s R correlation coefficient (**Supplementary Material**, **Supplementary Table S1**). Only variables with a significant association with functional outcome were then submitted to regression and mediation analyses.

One-way ANOVAs with center as the grouping factor were performed to evaluate significant differences between patients recruited at different participating sites on the following variables: age, education, psychopathology, TMT A, TMT B, Symbol Coding and FEIT. If the center effect was significant, the center was entered in the regression analyses.

To explore potential variables with the highest impact on functioning, we conducted three separate stepwise hierarchical multivariate regression analyses. Specifically, we used the PSP total score, the PSP score for socially useful activities and the PSP scores for personal and social relationships as dependent variables, and age, gender, years of education, center if its effect was significant on the ANOVA, positive dimension, disorganization, MAP, EXP, processing speed and emotion recognition as independent variables. In particular, the first regression module included age, gender, years of education and center, while the second regression module included positive dimension, disorganization, MAP, EXP, processing speed and emotion recognition.

**Figure 1:**
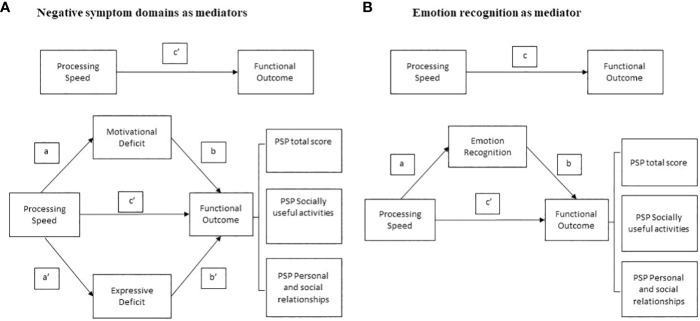
Basic model of mediation analyses with **(A)** negative symptom domains and **(B)** social cognition domain of emotion recognition as mediators between neurocognition and measures of functional outcome.

Mediation analyses were performed using PROCESS to determine the significance of the indirect effects (mediation) in six different models (**Figure 1**). For each model, the variable “processing speed” was considered as the independent variable or predictor, the two domains of negative symptoms (Motivational Deficit and Expressive Deficit) or social cognition were considered as mediator variables. The outcome variables, different for each model investigated, were 1) PSP total score, 2) PSP socially useful activities subscale and 3) PSP personal and social relationships subscale. To perform the mediation analyses, the predictor has to satisfy the assumption of a linear relationship with the outcome (**Figure 1** - path c); the mediator has to demonstrate a linear relationship with both the predictor (**Figure 1** - path a/a’) and the outcome (**Figure 1** - path b/b’). Partial mediation occurs when, after introducing the mediator variable into the model (negative symptom domains in **Figure 1A**; emotion recognition in **Figure 1B**), the direct effect (**Figure 1** - path c’) is reduced compared to the total direct effect (**Figure 1** - path c) but is still significant. Complete mediation occurs when the direct effect (**Figure 1** - path c’) is no more significant after introducing the mediator variable.

In our conceptual models (**Figure 1**), the effect of processing speed on functioning is referred to as the total effect (path c). In the three mediation analyses that considered negative symptom domains (Motivational Deficit and Expressive Deficit) as mediators (**Figure 1A**), the total effects included a direct effect of processing speed on functional outcome (path c’) and an indirect effect of processing speed on functioning through the two negative symptom domains (mediation paths: a/b, a’/b’). In the other three mediation analyses, in which social cognition was considered as a mediator (**Figure 1B**), the overall effects included a direct effect from processing speed to functional outcome (path c’) and an overall indirect effect from processing speed to functioning via social cognition (mediation path: a/b). The mediation analyses are considered valid when the significant level is maintained with 5000 bootstrapping samples and the 95% confidence interval of the indirect effect does not include zero.

All statistical analyses were performed using SPSS software version 25.

## 3 Results

### 3.1 Subjects

One hundred fifty subjects from 8 different European centers were recruited to the study. **Table 2** shows the demographic and clinical characteristics of the sample. The experimental sample consists predominantly of Caucasian (94%) and male (58%) subjects, with a mean age of 38.4 years, a mean duration of education of 13 years, and a mean duration of illness of 13 years.

**Table 2:** Demographic and clinical characteristics of the study sample.

	N	%	
Males	87	58	
Females	63	42	
Caucasian	141	94	
Others	9	6	
	N	Mean	SD
Age (yrs)	150	38.4	11.9
Education (completed yrs)	150	12.88	3.41
Duration of illness (yrs)	150	12.69	9.17
PANSS total score	150	61.87	17.49
PANSS positive	150	8.62	4.33
PANSS negative	150	12.75	5.43
PANSS disorganization (item P2)	150	2.21	1.31
BNSS total score	150	22.45	14.44
BNSS Motivational deficit	150	13.32	8.98
BNSS Expressive deficit	150	9.13	6.99
TMT A	139	46.7	30.7
TMT B	137	111.62	64.09
Symbol Coding	143	42.94	14.59
FEIT (number of correct answer)	82	36.13	9.09
PSP total	143	57.74	14.91
PSP Socially useful activities	143	2.84	1.26
PSP Personal and social relationships	143	2.31	1.13

SD, standard deviation; yrs, years; PANSS, Positive and negative syndrome scale; BNSS, Brief negative symptom scale; TMT, Trial making test; FEIT, Facial emotion identification test; PSP, Personal and social performance scale.

### 3.2 Regression analyses

The Center was entered in all regression analyses, as the ANOVAs showed a significant effect of the Center on age, psychopathology, functioning and symbol coding performance (**Table 3**).

**Table 3:** Center-effect for age, education, psychopathological and cognitive variables.

	C01	N	C02	N	C04	N	C05	N	C10	N	C11	N	C12	N	C17	N	*F*	*p*
**Age**	45 ± 12	25	35 ± 13	20	40 ± 10	20	30 ± 8.4	24	43 ± 8.9	25	39 ± 5.9	7	40 ± 12	20	32 ± 17	9	4.85	<.0001
**Education**	13.52 ± 3.95	25	14.37 ± 4.40	20	12.15 ± 2.48	20	12.25 ± 3.49	24	12.72 ± 3.07	25	12 ± 3.05	7	13.2 ± 2.84	20	11.44 ± 2.60	9	1.22	.271
**PANSS positive**	7 ± 4.20	25	8 ± 4.17	20	9.80 ± 4.65	20	10.88 ± 4.58	24	8.80 ± 4.97	25	8.57 ± 4.76	7	6.65 ± 2.23	20	9.78 ± 4.55	9	2.57	.016
**PANSS-P2**	1.92 ± 1.22	25	2.40 ± 1.35	20	2.95 ± 1.19	20	2.33 ± 1.31	24	2.32 ± 1.73	25	1.43 ± .79	7	1.74 ± .65	19	1.89 ± 1.05	9	2.10	.047
**BNSS Motivational deficit**	7.44 ± 6.73	25	12.1 ± 7.37	20	16.6 ± 7.41	20	11.29 ± 6.67	24	20.24 ± 10.22	25	15.57 ± 11.76	7	13.55 ± 9.48	20	9 ± 4.69	9	5.63	.00001
**BNSS Expressive deficit**	6.56 ± 5.90	25	10.15 ± 5.32	20	14.25 ± 6.09	20	6.37 ± 5.11	24	12.24 ± 8.48	25	9 ± 7.83	7	6.4 ± 7.06	20	7.44 ± 5.50	9	4.45	.00017
**PSP total**	58.92 ± 14.13	25	55.3 ± 15.06	20	45.25 ± 13.05	20	59.08 ± 10.83	24	61.84 ± 9.83	25	N/A	0	66 ± 21.08	20	54.33 ± 8.54	9	4.74	.00036
**PSP Socially useful activities**	3.72 ± 1.17	25	3.4 ± 1.19	20	3.2 ± .61	20	3 ± 1.14	24	2.08 ± .95	25	N/A	0	1.65 ± 1.27	20	2.67 ± .70	9	10.62	1.18x10^-9^
**PSP Personal and social relationships**	2.08 ± 1.08	25	3.1 ± 1.33	20	2.65 ± .67	20	2.63 ± 1.06	24	1.96 ± .89	25	N/A	0	1.7 ± 1.30	20	2 ± .86	9	4.43	.0004
**TMT A**	40.92 ± 19.51	25	49.25 ± 51.81	20	52.1 ± 28.62	20	30.95 ± 15.31	14	58.2 ± 30.28	25	38.83 ± 11.69	6	50.8 ± 29.26	20	33.78 ± 15.25	9	1.65	.127
**TMT B**	117.88 ± 77.99	25	113.44 ± 81.34	18	121.6 ± 44.55	20	83.26 ± 40.34	14	104.32 ± 51.43	25	102.17 ± 47.80	6	139.25 ± 78.42	20	77.78 ± 31.21	9	1.49	.177
**Symbol Coding**	43.16 ± 14.15	25	39.15 ± 13.22	20	47.05 ± 13.96	20	52.92 ± 10.07	24	31.4 ± 11.83	25	N/A	0	36.65 ± 8.32	20	61.11 ± 12.71	9	11.38	<.000001
**FEIT**	38.92 ± 7.08	25	38.75 ± 7.87	20	N/A	0	N/A	0	34.15 ± 10.22	20	N/A	0	31.29 ± 9.73	17	N/A	0	3.57	.180

PANSS, Positive and negative syndrome scale; BNSS, Brief negative symptom scale; TMT, Trial making test; FEIT, Facial emotion identification test; PSP, Personal and social performance scale.

The regression analyses demonstrated that the PSP total score was predicted by the expressive deficit domain of negative symptoms (β=-.430, p=1.3x10^-5^), and by the PANSS positive dimension (β=-.342, p=.001); the PSP socially useful activities score was predicted by the center (β=-.179, p=2.38x10^-14^), by processing speed (β=-.397, p=1.2x10^-4^) and by the motivational deficit (β=.270, p=.003), and the PSP personal and social relationships score was predicted by the gender (β=-.441, p=.028), by the center (β=-.109, p=2x10^-6^) and by the motivational deficit (β=.552, p=2.6x10^-7^) of negative symptoms (**Table 4**).

**Table 4:** Regression analyses to investigate factors that predict functional outcome.

		B	Std. Error	Beta	t	Sig.	95.0% Confidence Interval for B	
							Lower Bound	Upper Bound
**PSP total score**	Expressive deficit	-.430	.092	-.435	-4.65	1.3x10^-5^	-.614	-.246
	Positive symptoms	-.342	.099	-.325	-3.47	.001	-.539	-.146
**PSP Socially useful activities**	Center	-.179	.019	-.761	-9.36	2.38x10^-14^	-.217	-.141
	Processing Speed	-.397	.098	-.320	-4.048	1.2x10^-4^	-.592	-.202
	Motivational deficit	.270	.087	.259	3.113	.003	.097	.443
**PSP Personal and social relationships**	Gender	-.441	.197	-.201	-2.239	.028	-.833	-.049
	Center	-.109	.021	-.478	-5.165	2x10^-6^	-.151	-.067
	Motivational deficit	.552	.098	.542	5.645	2.6x10^-7^	.357	.746

B, unstandardized coefficient; Beta, standardized coefficient; PSP, Personal and social performance scale.

### 3.3 Mediation analysis

The following mediation analyses illustrate the role of negative symptoms or social cognition in mediating the effects of neurocognition on functioning.

#### 3.3.1 Negative symptoms as mediators between neurocognition and functional outcome

As reported in **Table 5**, the prerequisites of mediation analysis were not satisfied for the mediation role of negative symptom domains on PSP Socially useful activities.

**Table 5:** Statistics from the mediation analyses with negative symptom domains as mediators between neurocognition and functional outcome.

X	Y	M	N	a/a’, estimates (CI)	b/b’, estimates (CI)	c, estimates (CI)	c’, estimates (CI)	Indirect effect estimate (CI)
Model 1								
Processing speed	PSP tot	MAP	131	**-.342 (-.5477, -.136)**	**-.206 (-.39, -.013)**	**.274 (.078,.47)**	.119 (-.07,.31)	.704 (.0012,.1797) *
Processing speed	PSP tot	EXP	131	**-.372 (-.575, -.170)**	**-.227 (-.423, -.032)**			.085 (.0122,.1880) *
Model 2								
Processing speed	PSP SUA	MAP	131	**-.342 (-.5477, -.136)**	-.007 (-.18,.019)	**-.229 (.07,.47)**	-.18 (-.37,.01)	†
Processing speed	PSP SUA	EXP	131	**-.372 (-.575, -.170)**	-.13 (-.066,.032)			†
Model 3								
Processing speed	PSP PSR	MAP	131	**-.342 (-.5477, -.136)**	**.354 (.17,.53)**	**-.266 (-.46, -.07)**	-.098 (-.27,.080)	-.121 (-.231, -.045) *
Processing speed	PSP PSR	EXP	131	**-.372 (-.575, -.170)**	.12 (-.059,.31)			†

PSP, Personal and social performance scale; PSP SUA, PSP Socially useful activities; PSP PSR, PSP Personal and social relationships; MAP, Motivational deficit; EXP, Expressive deficit.

In boldface, p =< 0.05.

*Prerequisites to test for mediation are fulfilled, and the 95% CI of the indirect effect does not include zero and is therefore significant.

†Prerequisites to test for mediation are not fulfilled.

##### 3.3.1.1 Mediation analysis on PSP total score

**Figure 2:**
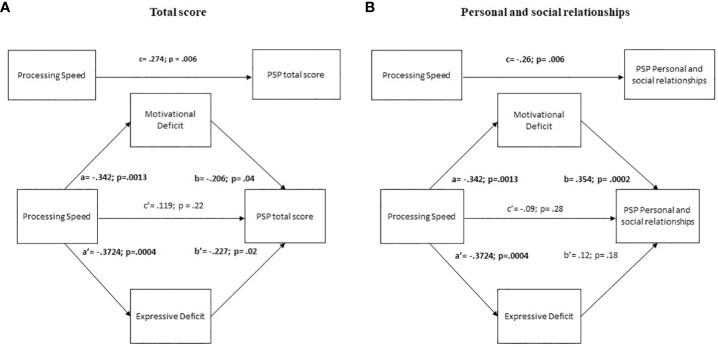
Mediation analyses with negative symptoms as mediator between neurocognition and the Personal and social performance scale (PSP) total score and Personal and social relationships. The negative symptom domains were both full mediators of the impact of Processing speed on PSP total score **(A)** while the motivational deficit domain was a full mediator of the impact of Processing speed on PSP Personal and social relationships **(B)**.

The results showed that the simple path coefficients (path a, b, a’, b’ and c’) were statistically significant at p<0.05 (**Table 5**; **Figure 2A**). The total effect of processing speed on PSP total score was.274 (p=.006), while the direct effect on the same score was no more significant, indicating a complete mediation of the effects by both negative symptom domains.

##### 3.3.1.2 Mediation analysis on PSP Personal and social relationships

The results showed that the simple path coefficients (path a, b and a’) were statistically significant (**Table 5**; **Figure 2B**). The total effect of processing speed on PSP Personal and social relationships was significant, while the direct effect was not indicating a full mediation effect through the motivational deficit domain of the negative symptoms.

#### 3.3.2 Social cognition as mediator between neurocognition and functional outcome

The results showed that the prerequisites were not satisfied for the mediation role of social cognition on PSP Personal and social relationships (**Table 6**).

**Table 6:** Statistics from the mediation analyses with social cognition as mediator between neurocognition and functional outcome.

X	Y	M	N	a, estimates (CI)	b, estimates (CI)	c, estimates (CI)	c’, estimates (CI)	Indirect effect estimate (CI)
Model 4								
Processing speed	PSP tot	ER	131	**.611, (.369,.854)**	**.231, (.006,.456)**	**.471, (.2273,.7144)**	**.330, (.054,.605)**	.141 (.022,.276) *
Model 5								
Processing speed	PSP SUA	ER	131	**.611, (.369,.854)**	**-.209 (-.415, -.004)**	**-.430 (-.653 -.208)**	**-.302 (-.554, -.051)**	-.128 (-.250, -.018) *
Model 6								
Processing speed	PSP PSR	ER	131	**.611, (.369,.854)**	-.098 (.432, -.347)	**-.338 (-.601, -.07)**	-.278 (-.582,.026)	†

PSP, Personal and social performance scale; PSP SUA, PSP Socially useful activities; PSP PSR, PSP Personal and social relationships; ER, Emotion Recognition.

In boldface, p =< 0.05.

*Prerequisites to test for mediation are fulfilled, and the 95% CI of the indirect effect does not include zero and is therefore significant.

†Prerequisites to test for mediation are not fulfilled.

##### 3.3.2.1 Mediation analysis on PSP total score

**Figure 3:**
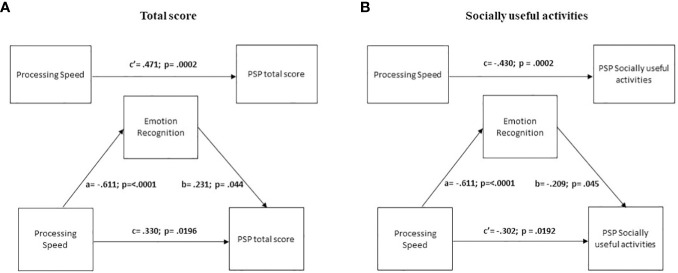
Mediation analyses with emotion recognition as mediator between neurocognition and the Personal and social performance scale (PSP) total score and Socially useful activities. The social cognition domain of emotion recognition partially mediates the impact of Processing speed on PSP total score **(A)** and of Processing speed on PSP Socially useful activities **(B)**.

The results showed that the simple path coefficients (path a, b, c and c1) were statistically significant (**Table 6**; **Figure 3A**). The total effect of processing speed on PSP total score was significant and moderate, while the direct effect was reduced though still significant. These results indicate a partial mediation effect of emotion recognition.

##### 3.3.2.2 Mediation analysis on PSP Socially useful activities

The results showed that the simple path coefficients (path a, b, c and c1) were statistically significant (**Table 6**; **Figure 3B**). The total effect of processing speed on PSP Socially useful activities was significant, and the direct effect was significant but reduced, again indicating a partial mediation effect of emotion recognition.

### 3.4 Control analyses

To rule out the possible confounding effects of positive symptoms on negative symptoms we conducted control analyses. Methods and results of these analyses are described within the **Supplementary Material**, **Supplementary Table S1**.

## 4 Discussion

Our data showed that positive symptoms and the expressive deficit domain of negative symptoms predicted global functioning. Furthermore, both negative symptom domains, the expressive deficit and the motivational deficit domain, fully mediated the effects of neurocognition on global functioning. The motivational deficit domain of negative symptoms was also a predictor of personal and social functioning and fully mediated neurocognitive impairment effects on the same outcome. Both motivational deficit and neurocognitive impairment predicted socially useful activities, which include work and everyday life skills, and the emotion recognition domain of social cognition partially mediated the impact of neurocognitive deficits on this outcome.

Our results are broadly consistent with previous literature showing a relationship between psychopathology, neurocognition and social cognition with functional outcome (4–6, 9, 15, 49, 63, 65, 67, 73, 76, 87–89). In particular, as regard to positive symptoms, even when they are of subclinical severity (mild severity in our sample), might contribute to global functioning. This is in line with previous literature findings (87–89). However, the global functioning, as evaluated by the PSP total score, includes psychopathological aspects and might thus explain why the positive symptoms are associated to this outcome measure but not to specific domains of real-life functioning which do not include any psychopathological aspect.

Among disease-related variables, as widely reported in the literature, cognitive deficits and negative symptoms seem to represent the major predictors of poor functional outcome (26–33).

The pathways to functional outcome are very complex especially when considering the large heterogeneity of negative symptoms, cognitive impairment and also functioning itself, which includes deficits in multiple domains (16, 26, 27, 90). Our results indicate the prominent impact of neurocognition on everyday life and work skills both directly and indirectly (trough social cognition) (6, 9) and the prominent role of the motivational deficit domain of negative symptoms on the interpersonal functioning domain (6, 9, 73).

Our study was designed to better understand the pathways from processing speed to functional outcome in individuals affected by schizophrenia (27, 37, 39–43).

Consistent with previous models (42, 48, 49, 91, 92), we found that both negative symptom domains mediated the relationship between processing speed and global functioning as assessed by the total score on PSP. It is worth noticing that the total PSP score includes both symptom severity and functional disability, probably explaining the predominant role of psychopathology, in particular negative symptoms, demonstrated by the regression and mediation analysis results of our study. When the PSP domains were considered, we found that the motivational deficit domain mediated the relationship between processing speed and PSP personal and social relationships. This finding is consistent with the evidence that the motivational deficit is associated with a greater impairment in functioning compared to the expressive deficit domain, particularly in the domain of interpersonal relationships (4, 6, 9, 19). Therefore, the use of different assessment tools, both for functioning and negative symptoms, could explain the heterogeneity in the findings reported by the literature.

Our results have clinical implications as the presence of negative symptoms, especially motivational negative symptoms might limit the transfer to the interpersonal functioning of cognitive remediation (16). The prevention and treatment of negative symptoms is thus mandatory and any effort should be made to control for the severity of factors that could induce negative symptoms, such as positive symptoms, extrapyramidal side effects or depression, which are the main sources of secondary negative symptoms (28). It is important to note that, in our study, the significant contribution of positive symptoms to global functioning raises the question whether negative symptoms in our sample were partly secondary to the positive symptoms. However, the sample was characterized by low levels of positive symptoms (absent to mild severity) and, in addition, correlation analyses between positive symptoms and the two negative symptom domains did not reach the statistical significance, thus excluding the possibility that the negative symptoms in our sample were secondary to the positive ones.

Regarding social cognition, we observed that it mediated the pathway from neurocognition to PSP Socially useful activities, suggesting that deficits in social cognition are influenced by processing speed (37), and interfere with real-life functioning in people with schizophrenia. These findings are consistent with a growing literature (93–96) in individuals with early psychosis (97) and with chronic schizophrenia (65, 98, 99). Other studies reported partially concordant results but found a relationship between social cognition and other neurocognitive domains, e.g., working memory, episodic memory, attention, problem solving, and executive functioning (97, 100–102). A possible interpretation of our results comes from the evidence that slower processing speed leads to greater cognitive effort in processing information, which could lead to deterioration in social cognitive skills. For example, due to a slower processing speed, and thus to a greater cognitive effort, a patient could experience communication conflicts due to errors in interpretation, resulting in difficulty recognizing the mental states of others and a loss of motivation. As a result, individuals with schizophrenia may have more negative experiences of social interactions, which can lead to feelings of low self-efficacy, greater problems with social skills and eventually increased social isolation (65).

This evidence could have some implications for clinical practice. For instance, the relationships found in the present study between social abilities and functioning highlights the importance to target also social cognition and not only neurocognition within functional recovery programs. In fact, meta-analyses have shown that social cognition can be successfully targeted by specific social cognitive remediation interventions (103, 104).

Furthermore, as indicated by the regression and mediation analyses, we did not find an association between social cognition and personal and social relationships, a finding that has been reported in previous studies on this topic (19, 27, 105). The absence of this relationship in the present study could be due to the relatively small proportion of subjects (82 out of 150) that participated in the social cognition assessment. In addition, the inconsistency between the results of our study and those of previous studies could be mainly due to two factors: i) most previous studies considered an overall score for social cognition and did not focus on a single domain of social cognition, whereas in the present study we focused on the domain of emotion recognition assessed by the FEIT; ii) the use of different assessment tools not only for social cognition but also for patients’ functioning.

Our results should be also interpreted in the light of some strengths and limitations.

First, few studies have attempted to investigate the possible role of negative symptom domains and emotion recognition as mediators in the relationship between processing speed and functional outcome using state of the art assessment instruments. Indeed, negative symptoms have been assessed using the BNSS, a second-generation scale that evaluates negative symptoms according to their current conceptualization (26). In addition, neurocognition was assessed with tests from the BACS, one of the brief instruments (27, 84, 106) recommended in the guidance paper on the assessment of cognitive deficits in patients with schizophrenia, issued by the European Psychiatric Association (27).

In terms of limitations, the sample size was relatively small, which limits the possibility of generalizing the results. In addition, there is also a very large effect of recruitment center, demonstrated by the results of one-way ANOVAs on key variables using the center as the grouping factor. Therefore, in the light of this effect, we kept the center as an explanatory variable in the regression models: if the center effect was found to be significant, we included “center” as a predictor in the regression models. Studies with larger samples and with a careful reduction of the recruitment center effect are needed to provide a further validation of these results. Another limitation of the present study may be related to the effects of pharmacological treatments that the patients underwent. However, as some meta-analyses on this topic have shown, psychotropic drugs do not seem to have a negative impact on processing speed (107) or on emotion recognition (108). Our study was a naturalistic unfunded study which demonstrated that using only a few indices of social cognition and neurocognitive functioning it is possible to better characterize in routine clinical settings patients with schizophrenia to personalize treatments (8).

The study of variables that may mediate and explain the relationship between cognition and functioning in people with schizophrenia is of great clinical and scientific importance. Our study focused on disease-related aspects, and in particular on cognition and negative symptoms, as they were found to be the key predictors of work, interpersonal and everyday life skills (6, 9). However, the significance of exploring non-disease-related factors as potential mediators between neurocognition and functional outcome should be further investigated. The intricate interplay between these variables merits further investigation to enhance our understanding of the multifaceted determinants of functional outcomes in schizophrenia. The development of early intervention programmes for psychosis could benefit from the identification of factors and pathways that influence functioning. It should be emphasized that combining approaches targeting neurocognition and social cognition with those targeting psychopathology offer synergistic benefits and may be essential for achieving long-term improvements in patient’ functioning (109–113).

## Data availability statement

The raw data supporting the conclusions of this article will be made available by the authors, without undue reservation.

## Ethics statement

The studies involving human participants were reviewed and approved by Ethical Committee from the 8 countries. The patients/participants provided their written informed consent to participate in this study. The studies were conducted in accordance with the local legislation and institutional requirements. 

## Author contributions

GG: Writing – original draft, Writing – review & editing, Conceptualization, Data curation, Methodology. PP: Writing – original draft, Writing – review & editing, Data curation, Methodology. AM: Supervision, Writing – original draft, Writing – review & editing, Conceptualization, Methodology. SA: Writing – review & editing. AE: Writing – review & editing. BG: Writing – review & editing. AH: Writing – review & editing. JH: Writing – review & editing. JL: Writing – review & editing. IM: Writing – review & editing. MN: Writing – review & editing. JR: Writing – review & editing. PW: Writing – review & editing. SG: Conceptualization, Supervision, Writing – original draft, Writing – review & editing. GS: Conceptualization, Supervision, Writing – original draft, Writing – review & editing.
