# Supplementary material for: Negative symptoms and social cognition as mediators of the relationship between neurocognition and functional outcome in schizophrenia
Source: Front Psychiatry. 2024 Jan 31;15:1333711. doi: 10.3389/fpsyt.2024.1333711 (PMC10864497; doi:10.3389/fpsyt.2024.1333711)
Supplement: Supplementary file 1 [file DataSheet_1.docx]

**Negative symptoms and social cognition as mediators of the relationship between neurocognition and functional outcome in schizophrenia**

**Index**

**1.** Correlation analyses

**1.1** Methods

**1. 2** Results of correlation analyses

**Table S1.** Correlations of demographic, clinical and cognitive variables with functional outcome.

**2.** Control analyses

**2.1** Methods

**2. 2** Results of correlation analyses

**3.** References

**1. Correlation analyses**

**1.1 Methods**

In order to evaluate the correlations of clinical and cognitive variables with functional outcome we performed correlation analyses, using Pearson's R correlation coefficient. Correlation coefficients between 0.10 and 0.29 in absolute value were interpreted as indicative of weak linear correlation, from 0.30 to 0.49 as moderate correlation, from 0.50 to 1 as strong correlation [1]. Results were corrected for multiple tests.

**1.2 Results** **of Correlation analyses**

Correlations between age, education, duration of illness, positive symptoms, negative symptom domains (motivational deficit and expressive deficit), disorganization, emotion recognition and processing speed with PSP total score, PSP socially useful activities and PSP personal and social relationships, are shown in table S1. The significance level for these analyses was set to p<.005 (p corrected for multiple tests).

The PSP total score showed significant moderate correlations with positive symptoms (r=-.382, p=3x 10^-6^), motivational deficit (r=-.371, p=5x10^-6^), expressive deficit (r=-.444, p=2.90x10^-8^), disorganization (r=-.392, p=1x10^-6^) emotion recognition (r=.325, p=.003) and processing speed; (r=.301, p=4.85x10^-4^); significant weak correlations with education (r=.287, p=5.11x10^-4^).

The PSP Socially useful activities showed significant moderate correlations with positive symptoms (r=.300, p=2.87x10^-4^), disorganization (r=.318, p=1.1x10^-4^) and emotion recognition (r=-.383, p=.001). Furthermore, this domain showed a weak correlation with the processing speed (r=-.244, p=.005), which survived correction for multiple tests and a weak correlation with expressive deficit (r=.222, p=.008) which did not survive correction for multiple tests.
Finally, the PSP Personal and social relationships showed significant moderate correlations with motivational deficit (r=.439, p=4.187x10^-8^), expressive deficit (r=.412, p=3.13x10^-7^), and disorganization (r=.350, p=2.5x10^-5^), and weak correlation with education (r=-.238, p=.004), positive symptoms (r=.278, p=7,64x10^-4^) and processing speed (r=-.268, p=.002).

**Table S1****. Correlations between demographic, clinical and cognitive variables with functional outcome**

|  | **PSP total score** | **PSP socially useful activities** | **PSP personal and social relationship** |
| --- | --- | --- | --- |
| **Age (yrs)** | -.049 | .136 | -.088 |
| **Education** | .287** | -.182 | -.238** |
| **Duration of illness** | -.144 | .139 | .050 |
| **Positive Symptoms** | **-.382**** | **.300**** | .278** |
| **Disorganization** | **-.392**** | **.318**** | **.350**** |
| **Motivational Deficit** | **-.371**** | .072 | **.439**** |
| **Expressive Deficit** | **-.444**** | .222* | **.412**** |
| **Emotion Recognition** | **.325**** | **-.383**** | -.159 |
| **Processing Speed** | **.301**** | -.244** | -.268** |

In **boldface** correlations with r≥0.30; * p<.05; ** p= < .005 (p value threshold corrected for multiple tests).
yrs = years; PSP = Personal and social performance scale

**2. Control analyses**

2.1. Methods

To rule out the possible confounding effect of positive symptoms on negative symptoms (secondary negative symptoms due to positive symptoms), we conducted correlation analyses between negative symptom domains and positive symptoms.

2.2. Results

We did not find any statistically significant correlation between positive symptoms and either the motivational deficit (r=.119, p=.145) or the expressive deficit (r=.134, p=.102) domain of negative symptoms.

**3.** References

1. Cohen, J., *A power primer.* Psychol Bull, 1992. **112**(1): p. 155-9.
